# A new species of *Zetekella* Drake from Ecuador with comments on *Zetekella* and *Minitingis* Barber (Heteroptera, Tingidae)

**DOI:** 10.3897/zookeys.796.23869

**Published:** 2018-11-15

**Authors:** Marcus Guidoti, Eric Guilbert

**Affiliations:** 1 Departamento de Zoologia, Universidade Federal do Rio Grande do Sul, Prédio 43.435, Av. Bento Gonçalves 9500, 91501-970, Porto Alegre-RS, Brazil Museum national d’Histoire naturelle Paris France; 2 UMR 7179 CNRS/MNHN, Museum national d’Histoire naturelle, CP50 – 57 rue Cuvier, 75005 Paris, France Universidade Federal do Rio Grande do Sul Porto Alegre Brazil; 3 Department of Entomology, National Museum of Natural History, Smithsonian Institution, 10th St. & Constitution Ave. NW, Washington, DC 20560, USA National Museum of Natural History, Smithsonian Institution Washington United States of America

**Keywords:** *Zetekella* Drake, Heteroptera, Tingidae, Ecuador, *Minitingis* Barber

## Abstract

*Zetekella* and *Minitingis* (Heteroptera, Tingidae) are morphologically similar genera, each comprising two species. The latter was already considered a junior synonym of the former, but was revalidated on the basis of the number of cephalic spines, projections on the paranotal edge, length of the rostrum, presence of an abdominal groove and distributional pattern. Here, a new species of *Zetekella* is described from Ecuador, the diagnoses for both genera reassessed, new records for *Z.pulla* and *Z.zeteki* reported, and a key to the species of both genera provided.

## Introduction

*Zetekella* Drake is composed of two species, *Z.zeteki* Drake, 1944 and *Z.pulla* Drake & Plaumann, 1956. After *Z.pulla* was described, the generic diagnosis was redefined, as follows: head moderately long to long, armed with five spines, bucculae open in front and slightly projected forward, and “rostrum extremely long, extending on venter” (Drake and Plaumann 1956). No macropterous forms are known for this genus, but other characters, such as the proportions of the antennal segments, often have been used in taxonomic studies of the Tingidae (excluding Vianadinae).

Zetekella was considered the senior synonym of *Minitingis* Barber by Drake and Ruhoff (1960) without further consideration of morphological characters or generic diagnoses. This genus was originally proposed to hold *Minitingisminusculus* Barber, 1954 on the basis of the number of pronotal carinae and the lateral acute processes of the paranota. However, the genus was compared with *Phatnoma*, rather than *Zetekella*, and the remarkable paranotal acute processes were found to vary by the same author (Barber 1954). Froeschner (1968) reinstated *Minitingis*, described a new species of the genus, and reaffirmed the generic status based on morphological characters and distributional patterns. According to Froeschner (1968), *Minitingis* could be distinguished by the presence of seven cephalic spines, the occipital pair being short and obliquely elevated, and the rostrum reaching the second abdominal segment. The paranotal development and the abdominal groove were also mentioned as diagnostic features of the genus (Froeschner 1968). Both *M.minusculus* and *M.elsae* Froeschner, 1968 are from the West Indies, whereas the known species of *Zetekella* are from Panama and Brazil. This distribution represents different zoogeographical zones and, therefore, corroborates the hypothesis of two genera (Froeschner 1968).

In this paper, we describe a new species of *Zetekella* from Ecuador, report two new records for *Z.pulla* and a new country record for *Z.zeteki*, and re-evaluate the diagnostic characters of both genera.

## Material and methods

### Material studied

The specimen here described was collected in a Berlese trap and had its abdomen removed for DNA extraction. The fixation method of the specimen is unknown, and it was preserved in 75% alcohol before the abdomen was removed and the specimen mounted. The specimen was point-mounted on the left side instead of the right side, to preserve two of its legs that accidentally had come in contact with the glue during the mounting process.

Holotypes of all species (except *M.minusculus*) were studied. For *M.minusculus*, a six-specimen series of paratypes was analyzed. All type material was examined at the National Museum of Natural History (**USNM**), in Washington, D.C., USA. Fifteen specimens of *Z.pulla* from the Museu de Zoologia da Universidade de São Paulo, Brazil, were also studied. The remaining specimens are housed in the first author’s personal collection.

### Species descriptions

Measurements of the holotype were taken from photos using ImageJ and are given in millimeters. Terminology follows the specialized literature (Drake and Davis 1960, Drake and Ruhoff 1965). The taxonomic act here treated was registered in Zoobank (Pyle and Michel 2008).

### Images

Photos were taken with a camera attached to a stereoscope and treated in GIMP. Plates were composed in Inkscape. The holotype photos of *Z.pulla*, *Z.zeteki*, and *M.minusculus* were kindly provided by Thomas Henry. Dorsal habitus and labels of the holotypes, voucher specimens for the new records, the two paratypes, and lateral and ventral views of the holotype of the new species were photographed and made available at Figshare.

### Keys

The keys to *Minitingis* and *Zetekella* species provided by Froeschner (1996) were merged, adapted and updated to include new species and new findings.

### Occurrence data

Geographic coordinates, when not available on the specimen labels, were obtained using Google Earth. The map was built using SimpleMappr (Shorthouse 2010). This map includes a layer with the Biodiversity Hotspots (sensu Conservation International; Mittermeier et al. 2004). Additionally, a spreadsheet containing occurrence data extracted from specimen labels was made available at Zenodo; the spreadsheet is organized by specimens and their unique identifiers, when available.

## Results

### Zetekella henryi sp. n.

Taxon classificationAnimaliaHemipteraTingidae

http://zoobank.org/733C8787-B04D-431F-B440-2EC04C13247B

Figs 1a , 2a

#### Material examined.

Holotype: ECUADOR, Orellana: Yasuni Research Station, 228m, 0.67°S, 76.40°W; 1–5 Dec 2009, D. Forero, EC09_L5, Berlese. MGPhD-E369. Male, Brachypterous.

#### Diagnosis.

Body dark brown to blackish; cephalic spines long and thin; anterior edge of paranota not reaching the eyes; discoidal area biseriate and subcostal area irregularly quadriseriate.

#### Description.

*Body* oval; mostly dark brown, or blackish; collar, paranota, and lateral edge of costal area and hemelytral membrane white; tip of cephalic spines, scape and pedicel light brown (basi- and distiflagellomere missing); occipital spines lighter in color.

*Head* with numerous, small, curved hairs and seven spines: clypeal pair non-erect; jugal spine slightly erect; frontal pair divergent; occipital pair short, strongly divergent; frontal and occipital pairs erect. Antenniferous processes spine-like, projected forward, subequal to scape in size. Scape slightly longer than pedicel, basi- and distiflagellomere missing. Interocular distance almost three times width of eye. Rostrum light brown, surpassing posterior margin of metanotum. Bucculae white, areolate; open in front, with an acutely projected antero-inferior edge; widely open posteriorly, width same as anterior region.

*Pronotum* mostly flat, posterior projection absent, leaving small portion of scutellum exposed. Median carinae whitish, uniseriate, composed of small cells, extending throughout pronotum. Collar biseriate and slightly elevated. Paranota slightly reflexed, broad, with four cells at widest part; anterior edge not reaching eyes. Sternal membranes whitish, areolate, uniseriate, and concave. Hemelytra ovate, inner border conspicuously concave posteriorly; clavus large, 2-seriate at widest part, inner vein straight, outer edge convex; discoidal area biseriate; cubitus whitish posteriorly after R+M junction; radius-media (R+M) white for most of length, raised, stout; subcostal area mostly 3-seriate, four rows of areolae at widest part; costal area wide, with as many as six rows of areolae, widening posteriorly; membrane shortened (specimen brachypterous); hypocosta dark brown, areolate anteriorly, but light brown, rim-like for most of length, ending at membrane. Scent-gland opening round, auricular-like, dark. Legs light brown, coxae and trochanters stout; longer, spine-like setae at posterior edge of tibiae; second tarsi long and slender. Claws long, slender, well developed.

*Pygophore* conspicuously narrower than abdomen; dorsal rim strongly curved, almost sinuous, forming small depressions laterally and dorsally. Paramere stout at base, abruptly but consistently narrowing to very slender tip, pronounced elbow at base.

*Measurements*: body length, 2.01; body width, 1.19; head length, 0.39; head width, 0.31; interocular width, 0.18; pronotum length, 0.35; pronotum width, 0.86; scape length, 0.06; pedicel length, 0.05.

#### Remarks.

Of the three known species of *Zetekella*, *Z.henryi* sp. n. is more morphologically similar to *Z.zeteki* because of the broader paranota and hemelytra, and the long clypeal, jugal and frontal cephalic spines. It differs from *Z.zeteki* by the thinner cephalic spines, the anterior edge of paranota not reaching the eyes, the narrower discoidal and subcostal area, and by its color pattern.

#### Etymology.

This species is named after the outstanding heteropterist and dear friend Thomas Henry, on the occasion of his 70^th^ birthday and his remarkable career and countless contributions to the study of Heteroptera.

**Figure 1.**
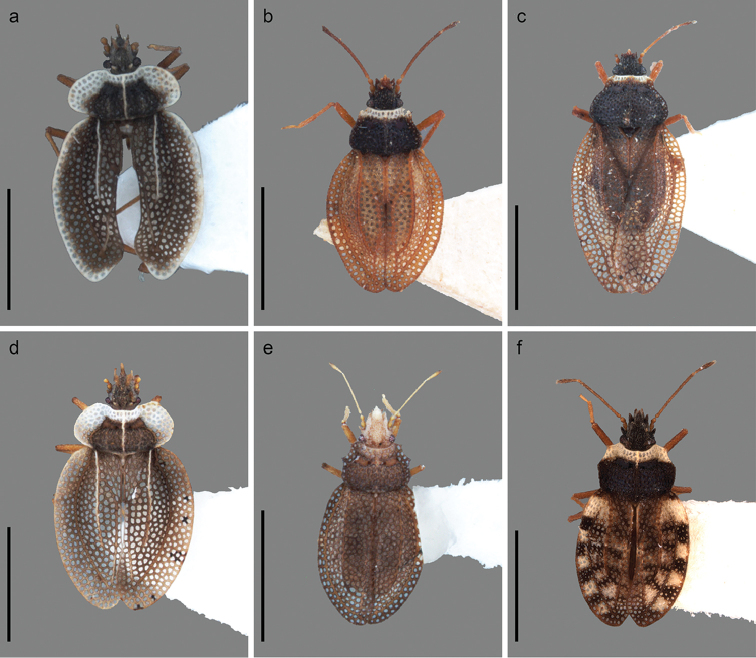
Dorsal habitus of *Zetekella* and *Minitingis* species. **a***Zetekellahenryi* sp. n. **b***Z.pulla*, brachypterous specimen **c***Z.pulla*, macropterous specimen **d***Z.zeteki***e***Minitingisminusculus***f***Minitingiselsae*. Scale bar: 1 mm.

**Figure 2.**
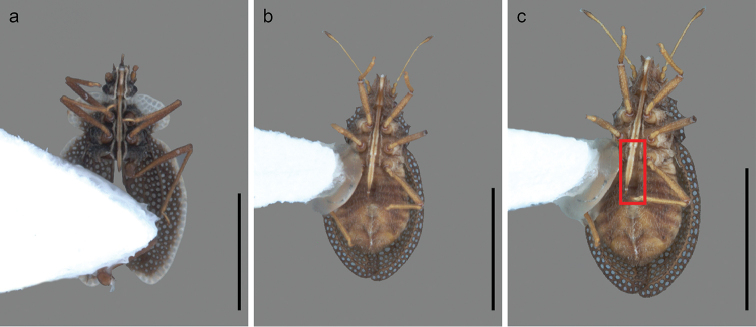
Rostral reach of *Zetekella* and *Minitingis* species. **a***Z.henryi* sp. n. **b***M.minusculus***c***M.minusculus* abdominal groove highlighted with a red square. Scale bar: 1 mm.

**Figure 3.**
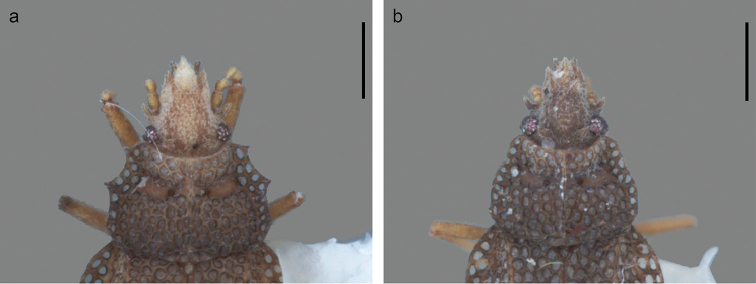
Variation observed in paranota of paratypes of *Minitingisminusculus*. Scale bar: 0.25 mm.

##### Key to *Zetekella* and *Minitingis*

**Table:** 

1	Rostrum conspicuously surpassing posterior edge of metathorax, reaching second or third abdominal segment, abdominal groove present	**2**
–	Rostrum surpassing posterior edge of metathorax, or not; not reaching second abdominal segment, abdominal groove absent	**3**
2	Costal area with alternate, conspicuous black and white quadrate marks, and 4 rows of areolae	***M.elsae*** (Fig. 1f)
–	Costal area without alternate black and white marks, and with 2 rows of areolae	***M.minusculus*** (Figs 1e, 2b, c, 3)
3	Paranota wide, with 4 to 5 rows of cells; costal area with at least 4 rows of cells	**4**
–	Paranota narrow, about half as wide as head, with 2 rows and a few cells irregularly placed; costal area with 2 rows of cells	***Z.pulla*** (Fig. 1b, 1c)
4	Body brownish, anterior edge of paranota reaching eyes, discoidal area mostly 3-seriate, subcostal mostly 4-seriate	***Z.zeteki*** (Fig. 1d)
–	Body dark brown or blackish, with collar, paranota, radius-media and lateral part of costal area and elytral membrane white, discoidal area mostly biseriate, subcostal irregularly quadriseriate	***Z.henryi* sp. n.** (Figs 1a, 2a)

## New records (Figure 4)

*Zetekellapulla*: BRAZIL. Santa Catarina: Ibicaré, 27°09, 51°18, 600m, F. Plaumann, Set. 1960. DZUP 387511-387515. **New record**. BRAZIL. São Paulo: Barueri, 23/VII/1967, K. Lenko - col. **New state record**.

*Zetekellazeteki*: COSTA RICA: Heredia: La Selva Biological Station, nr Puerto Viejo, clearing, 59m, 10.426946°N, 84.001449°W, 9–15 Aug 2010, OTS Heteroptera course [Berlese]. MGPhD-E290. **New country record** (Figure 1d).

**Figure 4.**
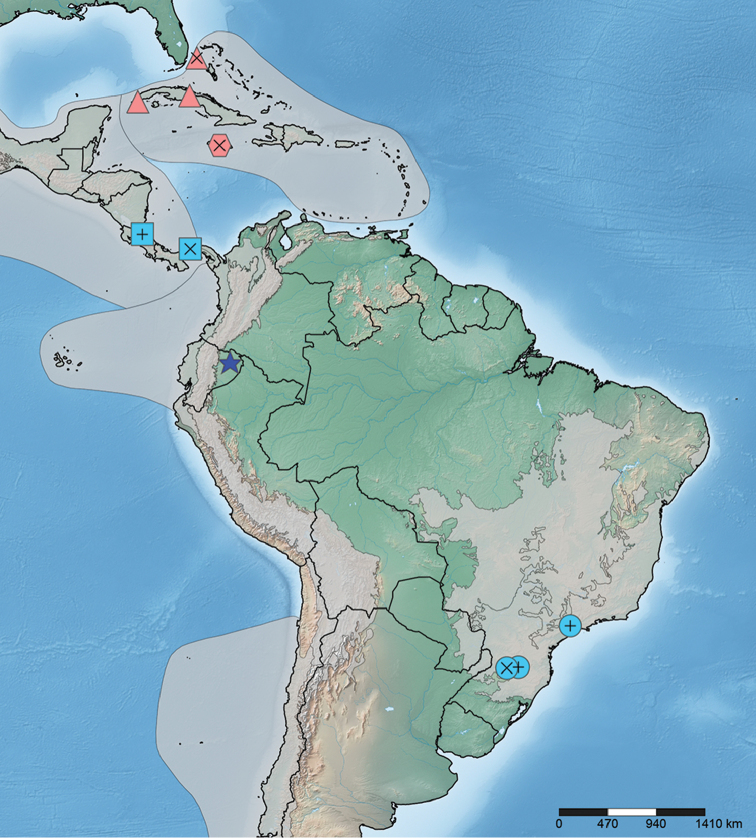
Distribution records for species of *Zetekella* and *Minitingis*. Blue icons = *Zetekella* species; square, circle, and star = *Z.zeteki*, *Z.pulla*, and *Z.henryi* sp. n., respectively; red icons = *Minitingis* records; triangle = *M.minisculus* and hexagon = *M.elsae*. Internal crosses = holotype localities; internal plus signs = new records.

## Data resources

SimpleMappr http://www.simplemappr.net/map/8595

KML http://www.simplemappr.net/map/8595.kml

Zoobank *Zetekellahenryi* n. sp.: 9480B3E7-E726-4718-8EBF-69C58A867887

Figshare Photographs of the dorsal habitus and labels of all holotypes (except *M.minusculus*), two paratypes of *M.minusculus* and of the new records vouchers

Zenodo Spreadsheet containing label information of all studied specimens and their respective unique identifiers

## Discussion

*Zetekellahenryi* sp. n. is described based on morphological differences in characters that have been commonly used to delimit species within Tingidae. The new species resembles *Z.zeteki*, but differs from it by the color pattern, paranota, and discoidal and subcostal areas of the hemelytra. Additionally, the shorter rostrum and shape of the scent gland allies these two species with *Z.pulla*. In addition to the description of a new species of *Zetekella*, a macropterous specimen of *Z.pulla* was found and is illustrated. All characters, except the hemelytral membrane, remain virtually the same between the macropterous and brachypterous specimens. Only brachypterous specimens previously have been known for species of *Zetekella* and *Minitingis*. We do not agree with the terminology typically used in the specialized literature to differentiate these two wing forms, but we reserve this subject for a more comprehensive, and illustrated, treatment in a future contribution.

Froeschner (1968) noted that only *Minitingis* and *Gonycentrum* Bergroth have seven cephalic spines in Phatnomatini, assuming that *Zetekella* has only five. Drake (1944), however, in describing the genus and *Z.zeteki*, already had observed that “there are indications of a pair of spines on the head behind the eyes and just in front of the collar” and that “as these are very much atrophied, they are not mentioned in the generic description.” Because the type specimen housed at the USNH is missing the head, this statement could not be verified. This feature, however, could be seen in the voucher specimen for the new record. Moreover, these spines were also observed in the new species. Yet, the mistake was perpetuated in the identification keys of Froeschner (1996). Froeschner (1968) also delimited and revalidated *Minitingis* on the basis of the acute processes of the paranota, which, however, can vary (Barber 1954).

In addition to cephalic spines and pronotal processes, Froeschner (1968) used rostrum length and presence of an abdominal groove as characters that validate the genus *Minitingis*. These characters were not possible to observe in the holotype (and single known specimen) of *M.elsae* due to the way the specimen is mounted, but they could be seen in all specimens of *M.minusculus* studied. We agree with Froeschner (1968) in regarding these two characters as reliable for distinguishing *Minitingis* from *Zetekella*. Froeschner’s (1968) comments on the zoogeographical significance of the distributional records of both genera remain relevant following our description of a new species of *Zetekella* and report of new distribution records for *Z.pulla* and *Z.zeteki*.

Therefore, we still consider *Minitingis* a valid genus, but we expanded the diagnosis of *Zetekella* to include the occipital cephalic spines and removed the acute processes on the paranota as a reliable character for delimiting *Minitingis*.

## Supplementary Material

XML Treatment for Zetekella henryi

## References

[B1] Alayo PD Grillo HR (1976) Los hemípteros de Cuba-XVII Redescubrimiento de la Chinche de encaje más rara de Cuba y nuevo reporte de otra especie afín (Hemíptera: Tingidae, Cantacaderinae). Centro Agrícola Sep-Dec: 112–116.

[B2] Barber HG (1954) A Report on the HemipteraHeteroptera from the Bimini Islands, Bahamas, British West Indies. American Museum Novitates 1682: 1–18.

[B3] Drake CJ (1944) Concerning the American cantacaderinids (Hemiptera: Tingitidae). Boletin de Entomologia Venezolana 3(3): 139–142.

[B4] Drake CJ (1950) Concerning the Cantacaderinae of the world (Hemiptera:Tingidae). Arthropoda 1(2–4): 153–166.

[B5] Drake CJ Davis NT (1960) The morphology, phyogeny, and higher classification of the family Tingidae, including the description of a new genus and species of the subfamily Vianaidinae (Hemiptera: Heteroptera). Entomologica Americana 39: 1–100.

[B6] Drake CJ Plaumann F (1956) A new cantacaderid from Brasil (Hemiptera: Tingidae). Bulletin of the Southern California Academy of Sciences 55(1): 17–18.

[B7] Drake CJ Ruhoff FA (1960) Lace-bug genera of the world (Hemiptera: Tingidae). Proceedings of the United States National Museum 112: 1–103. 10.5479/si.00963801.112-3431.1

[B8] Drake CJ Ruhoff FA (1965) Lacebugs of the world: A catalog (Hemiptera: Tingidae). United States National Museum Bulletin 243: 1–634. 10.5479/si.03629236.243.1

[B9] Froeschner RC (1968) Notes on the systematics and morphology of the lacebug subfamily Cantacaderinae. Proceedings of the Entomological Society of Washington 70: 245–246.

[B10] Froeschner RC (1996) Lace bug genera of the world, I: Introduction, subfamily Cantacaderinae (Heteroptera: Tingidae). Smithsonian contributions to Zoology 574: 1–43. 10.5479/si.00810282.574

[B11] Mittermeier RA Gil PR Hoffman M Pilgrim J Brooks TM Mittermeier CG Lamoreaux J da Fonseca GAB (2004) Hotspots Revisited: Earth’s Biologically Richest and Most Endangered Terrestrial Ecoregions. CEMEX, Mexico City, 390 pp.

[B12] Pyle RL Michel E (2008) ZooBank: Developing a nomenclatural tool for unifying 250 years of biological information. Zootaxa 1950: 39–50.

[B13] Shorthouse DP (2010) SimpleMappr, an online tool to produce publication-quality point maps. http://www.simplemappr.net [Accessed September 30, 2017]

